# Development of a Positive Youth Development Program: Promoting the Mental Health of Stressful Adolescents Using Principles of Problem Solving Therapy

**DOI:** 10.1100/tsw.2006.77

**Published:** 2006-03-28

**Authors:** Daniel T.L. Shek, Joyce T.W. Chow

**Affiliations:** Social Welfare Practice and Research Centre Department of Social Work Chinese University of Hong Kong, Shatin, Hong Kong

**Keywords:** youth development, mental health, Problem Solving Therapy, Chinese, Hong Kong

## Abstract

This paper outlines the proposal for the development, implementation, and evaluation of a positive youth development program that attempts to promote the mental health of stressful Chinese adolescents using principles of Problem Solving Therapy (PST). There are two general aims of PST: to help clients identify life difficulties and resolve them, as well as to teach them skills on how to deal with future problems. The proposed project will utilize the principles of PST as the guiding framework to run two mental health promotion courses for adolescents who are experiencing disturbing stressful responses and students who want to improve their stress management style. Both objective and subjective outcome evaluation strategies will be carried out to assess the effectiveness of the intervention to promote the psychological well-being in adolescents who are experiencing stress. A related sample proposal is described that can give social workers some insight on how to prepare a proposal for developing the Tier 2 Program of the Project P.A.T.H.S. (Positive Adolescent Training through Holistic Social Programs).

## INTRODUCTION

The Project P.A.T.H.S. (Positive Adolescent Training through Holistic Social Programs) is a positive youth development program designed to promote holistic adolescent development in Hong Kong, which is funded by the Hong Kong Jockey Club Charities Trust[1,2,3]. There are several characteristics of the Tier 2 Program of this project. First, the Tier 2 Program (Selective Program) targets adolescents with greater psychosocial needs who are identified in the Tier 1 Program and/or via other sources. It is recommended that information based on multiple sources, including objective assessment tools (e.g., Family Assessment Instrument, Life Satisfaction Scale, Hong Kong Student Information Form), teachers' ratings, student records, and other relevant quantitative and qualitative information based on systematic assessment, will be used to identify students for the Tier 2 Program. Second, it is suggested that particular attention should be paid to students with special needs in the academic, personal (e.g., adjustment, mental health, and value concerns), interpersonal, and family domains. As a general guideline, it is expected that at least one-fifth of the adolescents and/or the parents of the Tier 1 participants will participate in the Tier 2 Program.

Third, for students who are identified as having special needs, they and/or their families will receive services based on the Tier 2 Program. The NGO applicants have the choice of designing appropriate programs that target the needs of the students with reference to the positive youth development constructs, goals, and objectives covered in this project. Several nonmutually exclusive examples for the Tier 2 Program include: (1) mentorship programs involving the alumni of the schools, (2) mental health promotion programs, (3) adventure-based counseling, (4) parenting programs, (5) service learning programs, and (6) resilience enhancement programs. Fourth, NGOs will be invited to develop the Tier 2 Program with reference to the needs of the students and the positive youth development constructs covered in the program. The submitted proposals will be considered by the Social Welfare Department with the assistance of the Research Team.

Finally, the applicants have the choice of designing appropriate programs that meet the specific needs of the students. In the proposal, the applicants are expected to outline the project objectives, conceptual bases of the intervention model, rationales for using the model, and evidence supporting the effectiveness of the proposed approach.

## SAMPLE PROPOSALS TO PROMOTE ADOLESCENT PSYCHOLOGICAL WELL-BEING

In contrast to the Tier 1 Program, where the program was developed by the Research Team, agencies providing school social work services have to submit proposals for the Tier 2 Program. To facilitate workers to prepare quality proposals, the first author has prepared two sample proposals on the development, implementation, and evaluation of positive youth development programs. In this paper, a sample proposal on how to use the principles of Problem Solving Therapy (PST) to help stressful adolescents to improve their mental health is described. This sample proposal is included in the Guidance Notes on Applications for the P.A.T.H.S. Project as an illustration of the support provided to the applicants.

## STRESS IN CHINESE ADOLESCENTS

Adolescence has been conceived as a period of “storm and stress”. According to traditional Freudian concept, adolescence is a period that is tumultuous and emotionally unstable. Furthermore, with the changing socioeconomic conditions in the global context (e.g., intense pace of globalization) and local scene (e.g., persistently high adolescent unemployment rates), adolescents are facing more stressors. A review of the literature shows that Chinese adolescents are vulnerable to two major sources of stress.

The foremost stress faced by Chinese adolescents is academic stress. Historically, Chinese parents have regarded academic achievement to be very important because academic achievement was the basic means to climb up the social ladder. In the contemporary Chinese culture, many parents still hold the belief of “the sea of learning knows no bounds; only through diligence may its shore be reached” (xue hai wu yai, wei qin shi an). Many parents also regard diligence to be a key to successful academic performance, as exemplified in the saying that “reward lies ahead of diligence, but nothing is gained by indolence” (qin you gong, xi wu yi). Against such a background, academic stress has been found to be a major stressor faced by adolescents. For example, Shek and Chan[4] showed that having academic achievement was regarded by parents in Hong Kong as an important attribute of an ideal child.

There are many studies showing that Chinese adolescents in Hong Kong faced high academic stress. Chan[5] showed that making failing grades on a report card was regarded by 63% of the respondents as stressful. In the study of children and adolescent stress, the Wancahi Provisional District Board and City University of Hong Kong[6] showed that a high proportion of students showed worry about failure to attain ideal scores in examinations. The Hong Kong Federation of Youth Groups[7] found that roughly one-quarter of the respondents responded that getting further education was the greatest stress faced by them. In another study, the Hong Kong Federation of Youth Groups[8] showed that a significant proportion of the respondents displayed stress related to their study.

The second category of stress faced by adolescents is family stress. With the increase in economic independence of women and social acceptance of marital disruption, divorce rates and single-parent families have increased tremendously in the past 2 decades. With more people going back to the mainland to work, extramarital affairs have also become more prominent. Obviously, divorce and marital problems are stressful not just for the divorcees, but also for their children as well. Shek[9] showed that family stress in adolescents has increased in recent years.

Another area of family stress is related to the parent-child relationship. In a recent study examining the perceptions of family functioning among adolescents in Hong Kong, it was found that roughly one-third of the respondents perceived that their parents did not understand them, one-third to two-fifths of them had communication problems with their parents, and one-third of them regarded their families as stressful[10]. Under the influence of Confucian ideas, Chinese parents place great emphasis on filial piety. In Confucian thoughts, filial piety is the prime guiding principle in the socializing practices. The traditional saying that “among hundreds of behavior, filial piety is the most important one” (bai xing xiao wei xian) indicates that filial piety is highly valued by Chinese people. In particular, respect for the parents and obedience are highly valued. The traditional expectation for obedience is clearly revealed in the saying that “if a father wants the son to die, the son cannot disobey” (fu yao zi si, zi bu neng bu si). However, with the gradual breakdown of traditional Chinese family values and the demand for more freedom and involvement in decision making among Chinese young people, stress and conflict within the family are expected to increase.

## DISCUSSION

There are research findings showing that family stresses experienced by adolescents, such as family dysfunction, parent-child relational conflicts, and negative parent-child relations were negatively related to adolescent adjustment[11]. In their longitudinal study of the relationships between parental control processes and adolescent adjustment, Shek and his associates[12] also showed that negative parent-child relational qualities (e.g., lack of mutual trust between parents and their adolescent children and lack of readiness of adolescents to communicate with their children) were related to poorer adolescent psychological well-being.

With reference to the above background, it would be important to design programs that help stressful adolescents to deal with their stress. Although there are different approaches to stress management, the applications of PST can be regarded to be an evidence-based intervention approach. In this paper, an intervention program utilizing principles of PST is described and this proposal is included in the Guidance Notes on Application for the applicants. It is hoped that this sample proposal can help colleagues in the social welfare and education fields to develop Tier 2 Program for the P.A.T.H.S. Project.

## ACKNOWLEDGMENTS

This work was financially supported by the Hong Kong Jockey Club Charities Trust, Research Grants Council of the Government of the Hong Kong Special Administrative Region, Hong Kong (Grant CUHK4293/03H), and Wofoo Foundation.

## BIOSKETCHES

**Daniel T.L. Shek, PhD, FHKPs.S, BBS, JP**, is professor of Social Work in the Department of Social Work, The Chinese University of Hong Kong. He is on the editorial board of several scientific journals and has published extensively in international journals on social work, Chinese and English language, mental health, well-being, quality of life, adolescence, disadvantaged families, and drug abuse. E-Mail: DANIELSHEK@CUHK.EDU.HK. Website: http://www.cuhk.edu.hk/swk

**Joyce T.W. Chow**, **BSSC, RSW,** Social Welfare Practice and Research Centre, Department of Social Work, The Chinese University of Hong Kong, Hong Kong. E-mail: twchow@cuhk.edu.hk

## APPENDIX

A proposal on the development of a positive youth development program based on the application form for Project P.A.T.H.S. (Positive Adolescent Training through Holistic Social Programs) funded by the Hong Kong Jockey Club Charities Trust.

**1. Proposed program title**

Application of Problem Solving Therapy (PST) — Mental Health Program For Adolescents

**2. Aims of the program**

**2.1 Program aims:**

To promote the mental health of stressful adolescents using principles of PST

**2.2 Coverage of the 15 Positive Youth Development Constructs by the program**

Please copy this "☑" sign to the appropriate box(es).

☐ Promotion of Bonding
☑ Cultivation of Resilience
☐ Promotion of Social Competence
☑ Promotion of Emotional Competence
☑ Promotion of Cognitive Competence
☐ Promotion of Behavioral Competence
☐ Promotion of Moral Competence
☐ Cultivation of Self-Determination
☐ Promotion of Spirituality
☐ Development of Self-Efficacy
☐ Development of a Clear and Positive Identity
☐ Promotion of Beliefs in the Future
☐ Providing Recognition for Positive Behavior
☐ Providing Opportunities for Prosocial Involvement
☐ Fostering Prosocial Norms

**3. Specific objectives to be achieved**

1. To help adolescents to identify current life problems that are antecedents of maladaptive responses, such as a sense of hopelessness
2. To cultivate problem-solving skills that enable adolescents to resolve their life problems more effectively
3. To reduce maladaptive responses of the clients

**4. Proposed intervention model to achieve the project objectives**

The present project will utilize the principles of PST as the guiding framework to run two mental health promotion courses (D'Zurilla and Nezu, 1999; Nezu, Nezu, and Perri, 1989) for adolescents. Because there are cross-cultural differences in stress, coping, and problem solving, adaptation of the program content to suit the local scene and additional sessions will be designed (Satterfield, 2002; Shek, 2002). Construction of indigenous training materials will also be attempted.

**4.1 Conceptual bases of the intervention model**

Problem-solving therapy is a cognitive-behavioral approach to treatment and prevention of mental disorders (Steiman and Dobson, 2002). A review of the literature shows that cognitive-behavioral principles and programs have been widely used to treat and prevent adolescent emotional problems by different professionals (e.g., Compton, March, Brent, Albano, Weersing, and Curry, 2004; Possel, Horn, Groen, and Hautzinger, 2004), including the social work field (Thyer and Wodarski, 1998a, 1998b). With specific reference to the model advanced by D'Zurilla and Nezu (1999) and Nezu, Nezu, and Perri (1989), the assertions and principles in the PST model are as follows:

- In many psychological disturbances, a reduced ability on the part of the clients to manage and solve day-to-day problems is observed.
- The way individuals solve their problems (such as success or failure) in life determine their emotional states. Maladaptive emotional responses (e.g., hopelessness) would emerge if a person cannot solve his/her problems in an adequate manner.
- Emotional disturbances arise from deficiencies in any one or a combination of the five major components of problem solving: problem orientation, problem definition and formulation, generation of alternative solutions, decision making, and solution implementation and verification.
- Problem orientation (PO) includes five specific processes: problem perception, problem attribution, problem appraisal, perceived control, and time/effort commitment.
- Problem definition and formulation (PDF) includes the following elements: gathering relevant and factual information, understanding the problem, setting a realistic problem-solving goal, and reappraising the problem.
- Regarding generation of alternative solutions (GAS), the quantity principle, deferment-of-judgment principle and variety principle are involved.
- In the component of decision making (DM), the following elements are involved: anticipating solution outcomes, evaluating (judging and comparing) solution outcomes, and preparing a solution plan.
- Regarding solution implementation and verification (SIV), the following skills are focused on: execution of the solution plan, self-monitoring, self-evaluation, self-reinforcement, troubleshooting, and recycling.
- There are two general aims of PST: to help clients to identify life difficulties and resolve them, and to teach them skills to deal with future problems.
- The PST can be applied in the contexts of individual counseling, group counseling, and mental health promotion programs.

### 4.2 Rationales for using the model

There are several arguments for choosing the PST as a practice model to promote adolescent mental health:

1. The model has a strong theoretical foundation closely related to the work of D’Zurilla and Nezu (1999).
2. The use of social problem-solving training and therapy has increased in a rapid pace in recent years (Chang, D’Zurilla, and Sanna, 2004). Cantoni and Cantoni (1990) showed that the problem-solving model was one of the top three theories used by counselors.
3. Program and training materials are available (e.g., D’Zurilla and Nezu, 1999; Nezu, Nezu, and Perri, 1989).
4. There are research findings that provide support to the facts that problem solving affects one’s ability to cope with life stresses and that problem-solving training is an effective treatment or prevention method for different adolescent adjustment problems.

### 4.3 Evidence supporting the effectiveness of the proposed approach

- Tisdelle and St. Lawrence (1986) reviewed PST intervention studies and suggested that there was support for the intervention. However, they also pointed out the methodological weaknesses of the related studies.
- Based on a review of related intervention research in the field, Coleman, Wheeler, and Webber (1993) concluded that participants displayed cognitive gains after participating in the program.
- Kazdin, Siegel, and Bass (1992) reported that the use of the PST was associated with improvement in children’s overall morbidity and prosocial competence.
- Lochman (1992) included problem-solving training in an anger management program.

Results showed that compared to the control group, program participants had higher self-esteem and problem-solving skills at post-test.

- D'Zurilla and Nezu (1999) concluded that there were numerous intervention studies supporting the effectiveness of problem-solving therapy and training.
- Chang, D'Zurilla, and Sanna (2004) reviewed the social problem-solving theory, research, and training applied to children and adolescents, and concluded that the approach "holds promise for addressing critical issues facing the youth of America" (p. 167).
- In short, there are research findings supporting the effectiveness of the proposed approach, although some of the related intervention studies had methodological weaknesses.

## 5. Proposed implementation details of the program

### 5.1 Potential service targets of the program

Students who are experiencing disturbing stressful responses and students who want to improve their stress management style will be recruited. They will be identified in the Tier 1 Program and/or by the referral of the school social worker. Standardized assessment tools based on the responses of the students, teachers, and workers and/or their observations will be used in the recruitment process. The total number of clients to be served is 64 Secondary 1 students.

### 5.2 Proposed content of the program

- Four courses with 16 students in each group will be held.
- There will be 14 2-hour sessions with the following proposed content (D'Zurilla and Nezu, 1999)

| Session | Proposed Content |
|---|---|
| 1 | Initial structuring: introduces the goals, rationales, and general format of the training program, stress and coping in Chinese people. |
| 2 | Problem orientation: how to recognize problems, negative problem orientation, positive problem orientation, role of positive self-statements. |
| 3 | Use and control of emotions in problem solving: role of emotions in problem solving, how to use emotions to facilitate problem-solving effectiveness. |
| 4 | Emotional control: control of disruptive emotion, emotional expression in Chinese people. |
| 5 | Problem definition and formulation: gathering information, correcting distortions and misconception. |
| 6 | Understanding the problem, setting goals, getting at the "real" problem. |
| 7 | Generation of alternative solutions: major blocks, generating relevant solution alternatives. |
| 8 | Basic principles for generating alternative solutions: quantity principle, deferment-of-judgment principle, variety principle. |
| 9 | Decision making: rough screening of solution alternatives, anticipating and evaluating solution outcomes. |
| 10 | Making a solution plan: possibilities and blocks, real-life challenges. |
| 11 | Solution implementation and verification: solution implementation, self-monitoring. |
| 12 | Self-evaluation and self-reflection on solution implementation and verification. |
| 13 | Maintenance and generalization: rapid problem-solving model, consolidation of training effects. |
| 14 | Facilitation of maintenance and generalization of problem-solving performance |

## 6. Proposed evaluation mechanisms

Details of the required evaluation mechanisms can be seen in Section 6.1. Besides, an additional evaluation mechanism is proposed (see Section 6.2).

**6.1 Required evaluation mechanisms**

A. **Number of programs and participants —** Because there are 160 students in Secondary 1, 64 Secondary 1 students (i.e., two-fifths of the students) will be recruited to participate in the four courses, with 14 2-hour sessions in each course. For those who have missed some of the sessions (e.g., sickness), intensive make-up sessions will be provided.

B. **Evaluation of the Program by the participants** — Each participant will be invited to respond to a self-administered subjective outcome evaluation form designed by the Research Team after completion of the Program that assesses their satisfaction with the Program and the worker. For each session, each member will also be invited to fill out a brief client satisfaction form designed by the worker at the end of the meeting. The form will be modelled after existing client satisfaction evaluation forms used in the agency.

C. **Changes in the Program participants** — Each participant will be invited to respond to the evaluation form designed by the Research Team described in Part B above after completion of the program that assesses their perceptions of changes that take place after joining the program (i.e., subjective outcomes). Besides, assessment of the workers' perceptions of the program and changes in the participants will be carried out using the subjective outcome evaluation approach.

**6.2 Other evaluation mechanisms**

Besides subjective outcome evaluation, evaluation based on objective outcome evaluation will be made. The participant will respond to self-administered rating scales at pre- and post-test stages, assessing changes in (1) competence in identifying stressors that are creating maladaptive responses, (2) problem-solving competence, and (3) levels of maladaptive behavior.

1. **Objective 1 —** Self-constructed measures assessing the participants' ability to identify stressors that are causing problems and their perceived impacts would be constructed with reference to the available literature. Face validity of the instruments will be examined before use.
2. **Objective 2 —** The 25-item Chinese Social Problem Inventory (Siu and Shek, 2005a, 2005b) will be used to assess different components of the social process model, including Rational Problem Solving, Avoidance Style, Negative Problem Orientation, Positive Problem Orientation and Impulsiveness/Carelessness Style.
3. **Objective 3 —** A 10-item measure of hopelessness (Shek, 1993; Shek and Lee, 2005) will be used to assess the level of hopelessness in the participants.

**7. Documentation of the evaluation findings**

A brief report utilizing the Report Template designed by the Research Team that documents the following areas will be submitted:

1. The achievement of output level (outlined in Section 6.1.A)
2. The participants' perception of the program and the worker(s)(outlined in Section 6.1.B)
3. Evaluation findings based on the subjective outcome evaluation and other evaluation mechanisms (outlined in Section 6.1.C and 6.2)
4. Brief description of the contents and implementation of the program.
